# Early Life Adverse Environmental Exposures Increase the Risk of Uterine Fibroid Development: Role of Epigenetic Regulation

**DOI:** 10.3389/fphar.2016.00040

**Published:** 2016-03-01

**Authors:** Qiwei Yang, Michael P. Diamond, Ayman Al-Hendy

**Affiliations:** Division of Translation Research, Department of Obstetrics and Gynecology, Medical College of Georgia, Augusta University, AugustaGA, USA

**Keywords:** fibroid, endocrine disrupting compounds, histone modification, polycomb repressive complex, Trithorax group, developmental reprogramming

## Abstract

Uterine Fibroids [UF(s), AKA: leiomyoma] are the most important benign neoplastic threat to women’s health. They are the most common cause of hysterectomy imposing untold personal consequences and 100s of billions of healthcare dollars, worldwide. Currently, there is no long term effective FDA-approved medical treatment available, and surgery is the mainstay. The etiology of UFs is not fully understood. In this regard, we and others have recently reported that somatic mutations in the gene encoding the transcriptional mediator subunit Med12 are found to occur at a high frequency (∼85%) in UFs. UFs likely originate when a Med12 mutation occurs in a myometrial stem cell converting it into a tumor-forming stem cell leading to a clonal fibroid lesion. Although the molecular attributes underlying the mechanistic formation of UFs is largely unknown, a growing body of literature implicates unfavorable early life environmental exposures as potentially important contributors. Early life exposure to EDCs during sensitive windows of development can reprogram normal physiological responses and alter disease susceptibility later in life. Neonatal exposure to the EDCs such as diethylstilbestrol (DES) and genistein during reproductive tract development has been shown to increase the incidence, multiplicity and overall size of UFs in the Eker rat model, concomitantly reprogramming estrogen-responsive gene expression. Importantly, EDC exposure represses enhancer of zeste 2 (EZH2) and reduces levels of histone 3 lysine 27 trimethylation (H3K27me3) repressive mark through Estrogen receptor/Phosphatidylinositide 3-kinases/Protein kinase B non-genomic signaling in the developing uterus. Considering the fact that distinct Mediator Complex Subunit 12 (Med12) mutations are detected in different fibroid lesions in the same uterus, the emergence of each Med12 mutation is likely an independent event in an altered myometrial stem cell. It is therefore possible that a chronic reduction in DNA repair capacity eventually causes the emergence of mutations such as Med12 in myometrial stem cells converting them into fibroid tumor-forming stem cells, and thereby leads to the development of UFs. Advancing our understanding of the mechanistic role epigenetic regulation of stem cells plays in mediating risk and tumorigenesis will help in pointing the way toward the development of novel therapeutic options.

## Introduction

Exposure to environmental toxicants and toxins causes epigenetic changes that play a role in the development of disease (Cook et al., 2005; Walker and Ho, 2012; Yang et al., 2015b). Identifying changes in epigenomic marks (e.g., DNA methylation, histone modifications, non-coding RNAs) in affected tissues/cells is not always feasible in humans. Herein lies one of the challenges in making a direct connection between exposure-induced epigenetic changes and health outcomes in human populations. UFs, also known as uterine leiomyomas, are the most common pelvic tumors, occurring in nearly 70% of all reproductive-aged women (Al-Hendy and Salama, 2006; Bulun, 2013). It is the leading indication for hysterectomy with a conservative economic burden of about $34.4 billion/year in the US alone (Cardozo et al., 2012). These UFs cause severe symptoms such as heavy, irregular, and prolonged menstrual bleeding, anemia, pelvic pain, bowel and bladder dysfunction, infertility, recurrent abortion, and many obstetric complications such as preterm labor, obstructed labor necessitating cesarean section, fetal malpresentation, and fetal anomalies, as well as postpartum hemorrhaging (Sabry and Al-Hendy, 2012). These morbidities exert a tremendous toll on an individual’s overall health and well-being, impacting the quality of life of women of all ethnicities. Understanding mechanisms which regulate normal and aberrant myometrial cell function is paramount in the management of UFs. Therefore, development of effective, safe and inexpensive approach for the management of UFs is highly needed to improve the quality of life among those affected by UFs, but also in consideration of the significant impact UFs have in the context of public health (Sabry and Al-Hendy, 2012).

## The Role of Estrogen in Non-Genomic and Genomic Signaling of UFs

A striking feature of UFs is their dependency on the ovarian steroids estrogen and progesterone (Bulun, 2013). A number of experimental data suggests that estrogen stimulates the growth of UFs through ER α. The primary roles of estrogen and its’ receptor α in UFs growth are permissive in that they enable tissue to respond to progesterone by inducing expression of the progesterone receptor (Ishikawa et al., 2010).

The biological effects of 17β-estradiol are mediated by two isoforms of the ERs (ERα and ERβ). Hormone-activated ERs form dimers. Since the two forms are coexpressed in many cell types, the receptors may form ERα (αα) or ERβ (ββ) homodimers or ERαβ (αβ) heterodimers. Although ERs are widely expressed in different tissues types, some notable differences in their expression patterns occur. For instance, the ERα is found in endometrium, ovarian stromal cells, and breast cancer cells. ERs mediate the effects of 17β-estradiol under physiologic and pathologic conditions. ERs trigger 17β-estradiol-sensitive gene transcription by binding to specific estrogen response elements (i.e., genomic mechanism) (Hewitt et al., 2003; Gielen et al., 2007; Winuthayanon et al., 2014). In the absence of the estrogen hormone, ERs are largely located in the cytosol. The estrogen binds to the receptor, triggering a cascade of events, starting with the migration of the receptor from the cytosol into the nucleus; dimerization of the receptor; and subsequent binding of the receptor dimer to specific sequences of DNA known as estrogen response elements. The DNA/receptor complex then recruits other proteins that are responsible for transcriptional activation, which eventually alters target gene expression. ERs are also found within the cell nucleus, and both ER subtypes have a DNA-binding domain and can function as transcription factors to regulate gene expression (Burns and Korach, 2012).

Some ERs can be rapidly activated to downstream kinase cascades by exposure of the cells to estrogen (i.e., non-genomic mechanism; Bjornstrom and Sjoberg, 2002, 2005; Wong et al., 2002; Hofmeister et al., 2012). These so-called “non-genomic” effects are independent of gene transcription or protein synthesis and involve steroid-induced modulation of cytoplasmic or cell membrane-bound regulatory proteins. Estrogen can modulate regulatory cascades, such as MAPK, PI3K, and tyrosine kinases through non-transcriptional mechanisms. Furthermore, steroid hormone receptor modulation of cell membrane-associated molecules, such as ion channels and G-protein-coupled receptors, e.g., GPR30 has been shown in diverse tissues (Kelly et al., 2003; Prossnitz and Arterburn, 2015).

Both ER-evoked genomic and non-genomic effects originate from a unique signaling network (Bjornstrom and Sjoberg, 2005). A growing amount of evidence suggests that non-transcriptional signaling plays a pivotal role in the estrogen effect, which has clinical relevance, particularly in the development of UFs. Fibroids are common estrogen-dependent uterine tumors that cause significant morbidity for women and inflict a substantial economic impact on the US health delivery system (Al-Hendy and Salama, 2006). Our *in vivo* data in a mouse model demonstrates the ability of an adenovirus-expressing dominant-negative ER to arrest fibroid growth (Hassan et al., 2010). Taken together, cellular activities of estrogen and EDCs are the result of a combination of non-genomic and genomic actions via membrane and nuclear ERs-mediated signaling pathways.

## Epigenetic Modifications: PcG Proteins and TrxG Proteins

**FIGURE 1:**
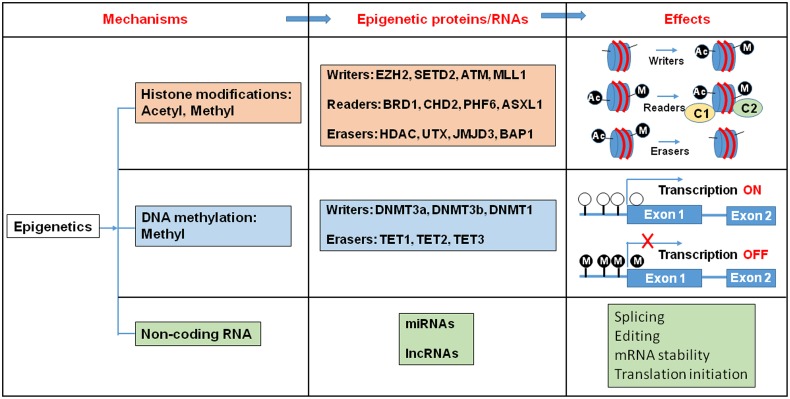
**Histone modification, DNA methylation, and non-coding RNA alter gene expression pattern.** Epigenetic writers catalyze the chemical modifications of amino acids on histones or the cytosine of DNA. Epigenetic erasers catalyze the removal of these modifications and epigenetic readers recognize the modifications and recruit large macromolecular complexes to the chromatin template. Ac, Acetyl; M, Metyl; C, protein complex.

**FIGURE 2:**
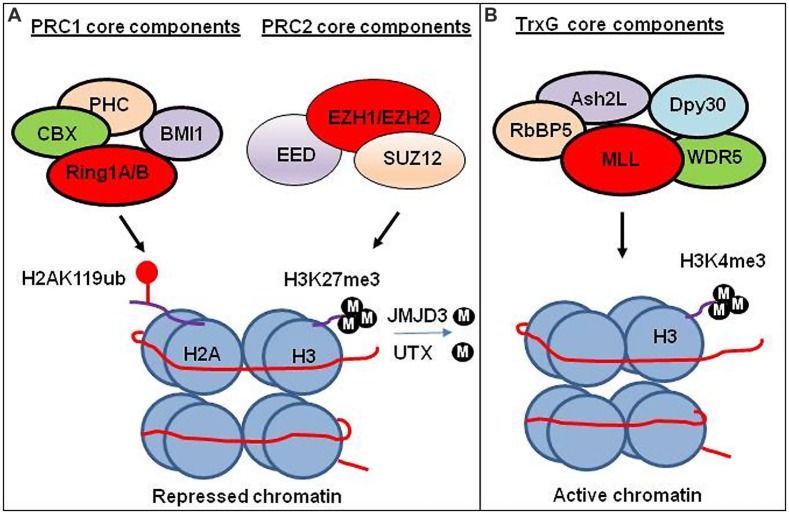
**Transcriptional regulation by PRC1, PRC2, and TrxG chromatin complex.** **(A)** Transcriptional regulation by PRC1 and PRC2 complex. PRC1 ubiquitinate H2A at lysine 119 (H2AK119ub). PRC2 trimethylates lysine 27 on histone 3 (H3K27me3). Experimental study suggests that H3K27me3 generated by PRC2 facilitates compaction of chromatin leading to the repression of gene expression. The CBX subunit of the PRC1 recognizes H3K27me3, and subsequently RING1A/1B subunits of the PRC1 ubiquitinate H2AK119 to facilitate the maintenance of the repressed state. H3K27 demethylases JMJD3 and UTX demethylate methylated H3K27. **(B)** Transcriptional regulation by TrxG complex. TrxG trimethylates histone 3 lysine (H3K4me3) leading to activate gene expression.

Epigenetic regulation is a dynamic process, which integrates environmental changes and enables cellular plasticity. As a result, it is involved in various pathologies related to environmental exposure to toxins. Proteins that carry out these epigenetic modifications are classified as “writers”, “readers”, and “erasers” (**Figure 1**). Epigenetic writers catalyze the addition of chemical groups onto either histone tails or onto the DNA itself (Cosgrove, 2012). These modifications are known as epigenetic marks (Vermeulen et al., 2010; Yun et al., 2011). Among them, PcG and TrxG proteins function as crucial epigenetic “writers” that regulate developmental gene expression in a variety of tissues and organs (**Figure 2**; Ringrose and Paro, 2007; Schuettengruber et al., 2007).

**FIGURE 3:**
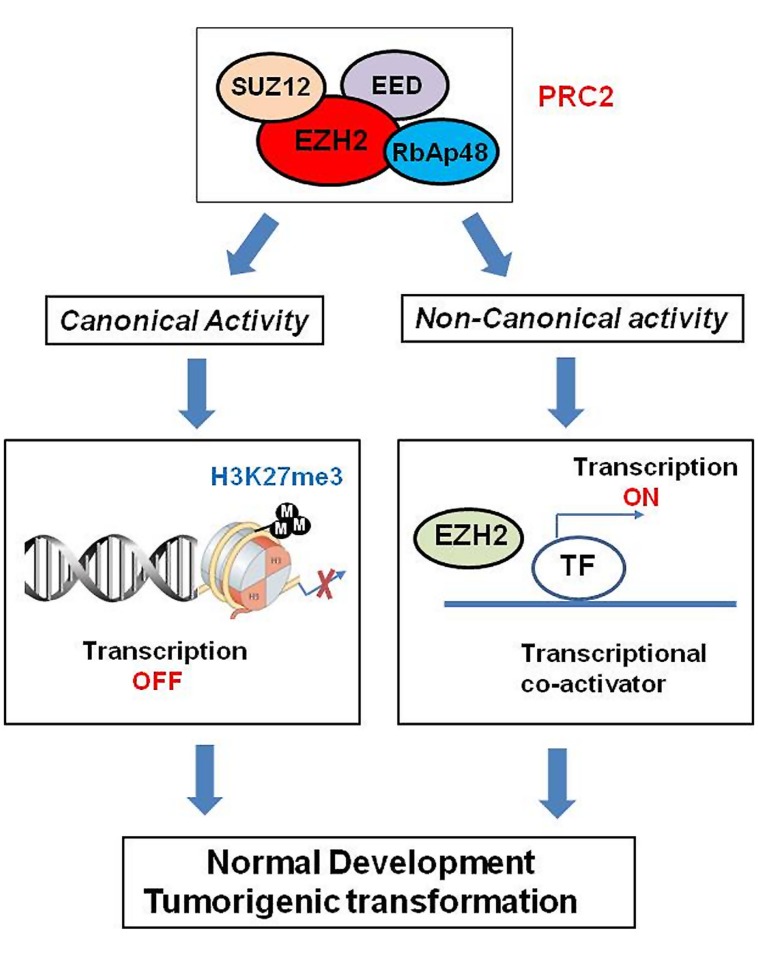
**Role of EZH2 in genomic signaling through canonical and non-canonical activity.** EZH2 confers long-term, heritable memory by sustaining silent gene expression states. In addition to its role as epigenetic modifier, EZH2 also works as transcriptional co-activators through non-canonical signaling pathway.

Polycomb group proteins form multimeric complexes that exert their functions by modifying chromatin structure and by regulating the deposition and recognition of multiple post-translational histone modifications (Morey and Helin, 2010). Two major PcG protein complexes have been described. The first complex, named polycomb repressive complex 1 (PRC1) is composed of four submits as shown in **Figure 2**. The second complex PRC2 consists primarily of EZH2, which is the catalytic core protein, EED, and SUZ12 (**Figure 2**). PRC2 methylates H3K27 via its EZH2 subunit. This modification, in turn, provides a binding site for the chromodomain-containing Pc subunit of PRC1, which subsequently leads to ubiquitination of H2AK119 via its Ring1a/1b subunit (Wang et al., 2004; Lehmann et al., 2012; **Figure 2**). In recent years, these proteins have raised considerable interest, due to their regulatory mechanisms and for the variety of key roles they play in normal cellular and disease processes (Villa et al., 2007; Pasini et al., 2010; Ntziachristos et al., 2012; Mozzetta et al., 2014; Serresi et al., 2016). For instance, EZH2 regulates chromatin structure and chromosome architecture at their target loci (**Table 1**) through canonical and non-canonical activity (**Figure 3**).

**Table 1:** List of EZH2-regulated genes.

Genes	Tumor type	Activation/repression	Journal	year
INK/ARF	Non-specific	Repression	EMBO J.	2003
RAD51	Breast	Repression	Neoplasia; Cancer Cell	2005; 2011
ADRB2	Prostate	Repression	Cancer Cell	2007
BMPR1B	Glioma	Repression	Cancer Cell	2008
CDH1	Prostate	Repression	Oncogene	2008
DKK1	Lung	Repression	Cancer Res.	2009
DARB2	Prostate	Repression	Nat. Med.	2010
VASH1	Ovarian	Repression	Cancer Cell	2010
CASZ1	Neuroblastoma	Repression	Cancer Res.	2011
CLU	Neuroblastoma	Repression	Cancer Res.	2011
RUNX3	Neuroblastoma	Repression	Cancer Res.	2011
NGFR	Neuroblastoma	Repression	Cancer Res.	2011
HOX	Mantle cell lymphoma	Repression	Epigenetics	2013
ITGA2	Colon	Repression	PloS ONE	2014
RUNX1	Prostate	Repression	Oncotarget	2014
MDR	Glioblastoma	Repression	Int. J. Clin. Exp. Pathol.	2014
MRP	Glioblastoma	Repression	Int. J. Clin. Exp. Pathol.	2014
BCRP	Glioblastoma	Repression	Int. J. Clin. Exp. Pathol.	2014
CDKN2A	Hepatocellar carcinoma	Repression	Mol. Cancer Res.	2014
E2F1	Hepatocellar carcinoma	Repression	Mol. Cancer Res.	2014
Notch2	Hepatocellar carcinoma	Repression	Mol. Cancer Res.	2014
TP53	Hepatocellar carcinoma	Repression	Mol. Cancer Res.	2014
E-cadherin	Renal	Repression	BJU Int.	2014
DCK	Melanoma	Repression	Nat. Commun.	2015
AMD1	Melanoma	Repression	Nat. Commun.	2015
WDR19	Melanoma	Repression	Nat. Commun.	2015
c-myc	Breast	Activation	Mol. Cell Biol.	2007
cyclinD1	Breast	Activation	Mol. Cell Biol.	2007
TNF	Breast	Activation	Molecular Cell	2011
IL6	Breast	Activation	Molecular Cell	2011
IL8	Breast	Activation	Molecular Cell	2011
AR	Prostate	Activation	Science	2012


*EZH2 is involved in regulation of gene expression through repressed or activated mechanism.*

Trithorax group proteins also function in multi-subunit complexes (**Figure 2**), confer heritable memory by sustaining active gene expression states, but they antagonize the function of the PcG (Schuettengruber et al., 2007, 2011; Steffen and Ringrose, 2014). For example, H3K4 trimethylation inhibits PRC2-mediated H3K27 trimethylation (Schmitges et al., 2011).

## Epigenetic Targets in Response to Environmental Factors in Some Tissues

The process of developmental programming exhibits a high degree of epigenetic plasticity, which is modifiable by intrinsic and extrinsic factors (Walker and Ho, 2012). However, when the *in utero* environment is suboptimal, permanent developmental reprogramming of the epigenetic targets could take place. Adverse environmental exposures during development can alter susceptibility later in life to adult diseases, including UFs (Cook et al., 2005, 2007; Greathouse et al., 2012). Increasing evidence suggests that early exposure to EDCs induces epigenetic changes in context to epigenetic regulated target genes in some tissues (Cook et al., 2005; Wong et al., 2015). For instance, neonatal exposure of CD-1 mice to EDCs such as DES (Walker and Ho, 2012; Gibson and Saunders, 2014), induces uterine adenocarcinoma in aging animals, concomitantly inducing hypomethylation of nucleosome binding protein1 (*Nsbp1*) promoter CpG Island (CGI) in the uteri which leads to persistent overexpression throughout life. Since the *Nsbp1* encodes a nuclear protein similar to the HMG 14, this protein may alter the gene expression pattern *in utero*, in response to early life EDC exposure leading to an increased risk of uterine cancer in adulthood (Tang et al., 2008). In rat mammary gland, prenatal exposure to BPA, another EDC, alters the epigenome and increases the propensity to neoplastic development. Accordingly, BPA exposure led to higher levels of MLL mediated epigenetic mark H3K4 trimethylation at the transcriptional initiation site of the alpha-lactalbumin gene, concurrently enhancing mRNA expression of this gene (Dhimolea et al., 2014). The protein encoded by this gene plays an important role in galactose metabolism. In addition, using a rat model for developmental reprogramming of susceptibility to prostate carcinogenesis (Yean Wong et al., 2015), neonatal exposure to BPA significantly upregulated (>100-fold) the expression of *Scgb2a1* in the prostate of adult rats via H3 lysine 9 acetylation. Importantly, Secretoglobin, Family 2A, Member 1 (*Scgb2a1)* encodes a component of prostatein, a major androgen-binding protein secreted by rat prostate, and hence suggests potential implications for cancer risk and response to chemotherapeutics associated with prostatein binding (Wong et al., 2015).

## Animal Model for Developmental Reprogramming of Susceptibility to UF Pathogenesis

Although there are several UF developmental models available (Hassan et al., 2009; Friel et al., 2010; Prizant et al., 2013; Mas et al., 2015), the best experimental animal model for studying UFs in response to early life adverse environmental exposure is the Eker rat model (Cook et al., 2005; Walker and Ho, 2012). Eker rats carry a germ-line mutation in the tuberous sclerosis complex-2 (*Tsc2*) tumor suppressor gene (Cook and Walker, 2004). In this Eker rodent model, the high spontaneous incidence of smooth muscle tumors of the uterus provides a unique opportunity to study the molecular mechanisms underlying the development of these clinically important neoplasms (Everitt et al., 1995). Using this model, Dr. Walker’s group demonstrated that early life exposure to EDCs including DES or genistein, a natural isoflavone phytoestrogen found in soybeans, increased tumor penetrance (from 65% to >90%), tumor multiplicity and overall size (Cook et al., 2005; Greathouse et al., 2012). This increased penetrance induced by early life environmental exposure to EDCs is associated with the reprogramming of estrogen-responsive genes, which become hyper-responsive to the estrogen hormone and promote the development of hormone-dependent UFs (Greathouse et al., 2008; Walker and Ho, 2012).

## Role of Epigenetic “Writers” in UF Development

Estrogen triggering genomic signaling in context to epigenetic “writers” has recently been identified. Bhan et al. (2014) demonstrate that EZH2 is transcriptionally induced by estradiol in cultured breast cancer cells and in the mammary glands of ovariectomized rats. Similar to estradiol, DES-induced EZH2 expression is coordinated by ERs, MLLs and CBP/P300. These studies suggest that EZH2 is potentially dysregulated upon exposure to EDCs, and provides a direct link between EDC-induced nuclear hormone receptor signaling and modulation of the epigenetic machinery (Bhan et al., 2014).

Until recently, little information has been available about the role of PcG/TrxG proteins in the development UFs. However, Dr. Walker’s group reported that DES is capable of binding to membrane-associated ER to activate non-genomic ER signaling, activating PI3K signaling and the kinase AKT. Subsequently Phosphorylation of serine 21 of EZH2 by AKT inactivates EZH2 leading to reduced levels of the repressive trimethylation of H3K27 in the developing uterus (Bredfeldt et al., 2010). A further study indicated that yet another environmental estrogen, genistein, also induced PI3K/AKT non-genomic ER signaling to the histone EZH2 (Greathouse et al., 2012). These studies demonstrate the importance of the interplay between non-genomic signaling and epigenetic mechanisms in response to early life environmental exposure to estrogen that may contribute to an increased risk of UF development.

## DNA Damage Repair in Stem Cells

**FIGURE 4:**
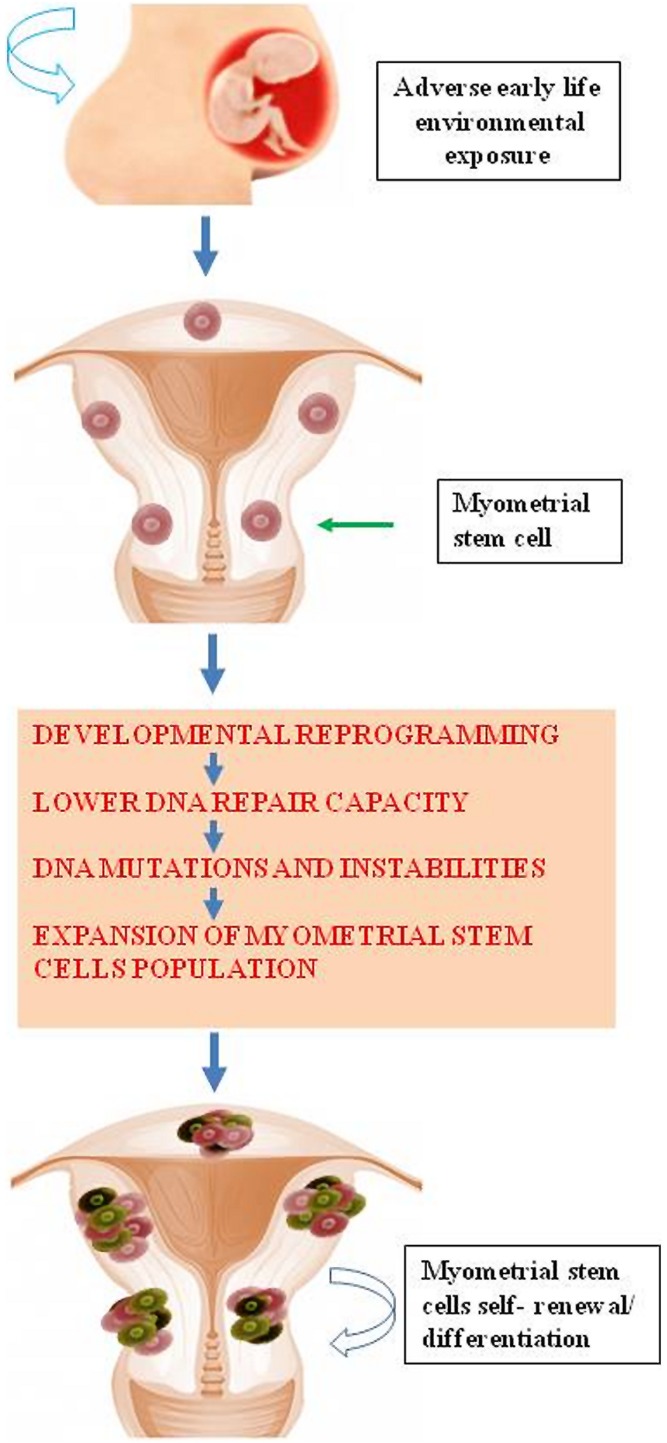
**Early life adverse environmental exposure compromises DNA damage repair system in myometrial stem cells through epigenetic regulation eventually leading DNA instability and mutations and subsequent formation of UFs**.

Accumulating evidence demonstrates that environmental chemicals or their reactive intermediates can react with DNA to modify DNA bases leading to DNA damage (Linder, 2012; Moller et al., 2013). Exposures can act through an epigenetic mechanism by which DNA damage repair is altered (Langie et al., 2013). Currently, Med12 somatic mutation is the most widely detected DNA mutation in human fibroid lesions. We and others have detected a single nucleotide Med12 mutations in up to 85% of sporadic fibroid lesions (Makinen et al., 2011a,b; Markowski et al., 2012; McGuire et al., 2012; Halder et al., 2014). Interestingly, distinct Med12 mutations are detected in different fibroid lesions in the same uterus (Makinen et al., 2011b). This strongly suggests that the emergence of each Med12 mutation is an independent event in an altered myometrial stem cells. It is possible that some risk factors attenuate key DNA damage repair gene function leading to reduced myometrial DNA repair capacity. This reduction in the DNA repair capacity may eventually cause the emergence of mutations such as Med12 in myometrial stem cells converting them into fibroid tumor-forming stem cells; and thereby, leading to the development of UFs (**Figure 4**). In a mouse model that conditionally expresses a Med12 missense variant (c. 131G > A), it has been demonstrated that this alteration alone promotes fibroid formation and drives genomic instability (Mittal et al., 2015).

A comparative analysis of dysfunctional DNA repair capacity in stem cells from fibroid tissues or at-risk myometrium with fibroid versus stem cells from normal myometrium has not yet been conducted. However, Chang et al. (2011) reported that BTICs exhibited increased EZH2 expression which was linked to decreased expression of key DNA repair gene *RAD51*. Therefore, accumulation of recurrent Raf-1 proto-oncogene, serine/threonine Kinase *(RAF1)* gene amplification in BTICs occurred, which activates p-ERK-β-catenin signaling to promote BTIC expansion (Chang et al., 2011). It has been shown that both human myometrial and UF tissues contain side population (SP) cells with progenitor/stem cell properties (Ono et al., 2007, 2012, 2013; Mas et al., 2012). We recently isolated human surface marker-specific myometrial and fibroid stem cells. Using Stro-1/CD44 surface markers, we were able to isolate stem cells from adjacent myometrium and human fibroid tissues using the magnetic beads approach (Mas et al., 2015). *In vitro* Stro-1^+^/CD44^+^ myometrial cells exhibit the ability to differentiate into adipocytes, osteocytes, and chondrocytes with the functional capacity to form fibroid-like lesions in a xenotransplantation mouse model. In the future, we will compare the DNA repair capacity of Stro-1^+^/CD44^+^ myometrial stem cells from normal human myometrium versus at-risk myometrium tissues or fibroids.

## Cell-Derived Exosomes: Roles in Tumor Development and Progression

Emerging evidence consistently demonstrates that exosomal miRNAs can be reprogrammed by environmental factors (Goustard-Langelier et al., 2013; Shah et al., 2016). Exosome are cell-derived small sized vesicles (40–150 nm), present in many biological fluids (Pan et al., 1985; Simons and Raposo, 2009). Exosomes are either released from the cells when multivesicular bodies fuse with plasma membrane or released directly from the plasma membrane. Emerging evidence indicates that exosomes contain a range of biological molecules, including mRNA, microRNA, long non-coding RNAs, proteins, lipids, molecular chaperones, and signaling molecules (Skog et al., 2008), as well as involvement in many biological events including cancer progression (Kogure et al., 2011; Luga et al., 2012). Importantly, the molecular signatures of exosomes are specific to each tissue type, providing an alternative option for clinical applications (Nawaz et al., 2014).

Exosomes exhibit fundamental paracrine mechanisms that mediate cell-to-cell communication and play a role in the transfer of messages from one cell to another (Simons and Raposo, 2009; O’Brien et al., 2013). Exosomes are important players in the regulation of physiological as well as pathological processes in our body - depending on their content, they can induce activation, proliferation, differentiation, or apoptosis of the recipient cells (Roma-Rodrigues et al., 2014; Ung et al., 2014). In cancer, this cell-to-cell communication leads to increased proliferation, motility, induction of invasive properties of the recipient cells, as well as conferring drug resistance (Kahlert and Kalluri, 2013).

Although the role of exosomes in tumor development is not well understood, some studies have highlighted a possible role in tumor development and progression. Exosomes with a specific surface protein (glypican-1) were found to be detected in the serum of patients with pancreatic cancer, distinguishing healthy subjects from those with benign pancreatic disease (Melo et al., 2015). Melanome exosomes educate bone marrow progenitor cells toward a pro-metastatic phenotype via MET, and exosome-mediated transfer of the oncoprotein. MET functions as a key regulator of bone marrow education, mobilization, and metastatic progression (Peinado et al., 2012). The exosomes from normal and abnormal cells differ in their cargo content, and potentially in their functions. For instance, breast cancer exosomes perform cell-independent miRNA biogenesis and alter the transcriptome of receipt cells in a Dicer-dependent manner (Melo et al., 2014).

Uterine Fibroids are thought to be monoclonal tumors arising from the myometrium, and tumor stem cells are considered to play pivotal roles in the tumorigenesis of UFs. It is possible that cell-to-cell interaction between myometrial stem cells and differentiation cells is involved in the development of UFs. Although the role of myometrial stem cell-derived exosomes is unknown, increasing, studies have suggested that stem cell-derived exosomes containing important effectors of Wnt (Luga et al., 2012), Hedgehog (Gradilla et al., 2014), and β-catenin (Chairoungdua et al., 2010), may play a potential role in maintaining stem cell characteristics. Cancer stem cell-derived exosomes contain distinct biomolecules as compared to exosomes derived from normal stem cells indicating the important role of exosomal miRNA content from cancer stem cells in cancer progression and development. For instance, gastric cancer tissue-derived mesenchymal stem cells favor gastric cancer progression by transferring exosomal miRNAs to gastric cancer cells and promote their proliferation and migration (Wang et al., 2014). Similarly, glioma-associated stem cells produce substantial amounts of exosomes which leads to sustained malignant properties of both glioma cells and glioma stem cells. Moreover, a recent study has shown that exosomes from bone marrow-derived mesenchymal stem cells transport tumor regulatory miRNAs, anti-apoptotic proteins, and metabolites that promote breast tumor growth (Vallabhaneni et al., 2015). These studies suggest that stem cell-derived exosomes contain important molecules that promote tumor progression.

The importance of stem cell-derived miRNAs in response to environmental factors has recently been identified (Goustard-Langelier et al., 2013; Shah et al., 2016). Shah et al. (2016) determined the effects of the chemoprotective fish oil/pectin diet on miRNAs in colonic stem cells obtained from Lgr5-EGFP-IRES-creER knock-in mice. They demonstrated that 26 miRNAs were differentially expressed in Lgr5 (high) stem cells as compared to Lgr5 (negative) differentiated cells. Fish oil/pectin treatment up-regulated miR-19b, miR-26b and miR-203 expression as compared to corn oil plus cellulose (CCA) specifically in Lgr5 (high) cells. They further demonstrated that only miR-19b and its indirect target PTK2B were modulated by the fish oil/pectin diet in Lgr5 (negative) cells. In addition, rat neural stem cells/neural progenitors (NSC) proliferation and differentiation were dually altered by the *in utero* polyunsaturated fatty acid supply, along with marked alterations in miRNA expression (Goustard-Langelier et al., 2013). Although the role of exosomes in the pathogenesis of UFs is unknown, we recently isolated myometrium stem cells from adult uteri early life exposed to DES. These cells will serve as a tool in determining how early life environmental exposure alters stem cell derived exosomal cargo and thereby leads to an increased risk of UF pathogenesis.

## Concluding Remarks

Currently, there is a remarkable lack of knowledge regarding the involvement of chromatin assembly in the process by which adverse environmental exposures increase the overall risk of UF development. The precise mechanism underlying EDC-dependent effects on myometrial cell physiology are not adequately understood. Accordingly, in response to EDC administration, no single PcG or TrxG-target genes have been discovered in myometrium tissues as well as in myometrial stem cells (Yang et al., 2015a). In addition to EZH2 “writer”, many other epigenetic proteins that play a role in UF development, need to be investigated. High throughput epigenetic analysis such as ChIP-seq are needed to determine locus specific and/or genome-wide epigenetic modifications in myometrial stem cells and tissues. A better understanding of these changes in myometrial stem cells will lead to the mechanistic plausibility as to the role of epigenetic regulation in mediating risk and tumorigenesis and the development of new stem cell-directed therapies for patients with UFs.

## Author Contributions

QY and AA, conception of the manuscript; QY, drafted the manuscript; QY, MD, and AA, revised the manuscript. All authors approved the revised version of the manuscript.

## Conflict of Interest Statement

The authors declare that the research was conducted in the absence of any commercial or financial relationships that could be construed as a potential conflict of interest.

## Abbreviations

AKT: Protein kinase B
Ash2L: absent, small, or homeotic-like
BMI1: B-B-lymphoma Mo-MLV insertion region 1 homolog
BPA: Bisphenol A
BTIC: breast tumor initiating cells
CBP: CREB binding protein
CBX: chromobox homolog
DES: diethylstilbestrol
Dpy30: dosage compensation-related protein 30
EDCs: endocrine disrupting compounds
EED: embryonic ectoderm development
ERs: Estrogen receptors
EZH2: enhancer of zeste 2
HMG 14: high mobility group protein 14
MAPK: mitogen-activated protein kinases
Med12: Mediator Complex Subunit 12
MET: Mesenchymal epithelial transition factor
MLL: mixed-lineage leukemia protein
P300: E1A binding protein P300
PcG: Polycomb group
PHC: polyhomeotic homolog
PI3K: phosphatidylinositide 3-kinases
RbBP5: retinoblastoma binding protein 5
RING1A or RING1B: ring finger 1A or 1B
SUZ12: Suppressor of zeste 12
TrxG: trithorax group
UFs: Uterine fibroids
WDR5: WD40 repeat domain 5
